# Chemokine CXCL13 is overexpressed in the tumour tissue and in the peripheral blood of breast cancer patients

**DOI:** 10.1038/sj.bjc.6604621

**Published:** 2008-09-09

**Authors:** J Panse, K Friedrichs, A Marx, Y Hildebrandt, T Luetkens, K Bartels, C Horn, T Stahl, Y Cao, K Milde-Langosch, A Niendorf, N Kröger, S Wenzel, R Leuwer, C Bokemeyer, S Hegewisch-Becker, D Atanackovic

**Affiliations:** 1 Department of Oncology/Hematology, Center of Oncology, University Medical Center Hamburg-Eppendorf, Martinistrasse 52, Hamburg 20246, Germany; 2 Mammazentrum Hamburg, Moorkamp 2-6, Hamburg 20357, Germany; 3 Institute for Pathology, University Medical Center Hamburg-Eppendorf, Martinistrasse 52, Hamburg 5220246, Germany; 4 Department of Stem Cell Transplantation, University Medical Center Hamburg-Eppendorf, Martinistrasse 52, Hamburg 20246, Germany; 5 Department of Gynecology, University Medical Center Hamburg-Eppendorf, Martinistrasse 52, Hamburg 20246, Germany; 6 Laboratory for Pathology Hamburg-Altona, Lornsenstrasse 4, Hamburg 22767, Germany; 7 Department of Otorhinolaryngology, University Medical Center Hamburg-Eppendorf, Martinistrasse 52, Hamburg 20246, Germany; 8 Private Practice for Oncology, Eppendorfer Landstrasse 42, Hamburg 20249, Germany

**Keywords:** chemokines, CXCL13, breast cancer, tumour expression, serum markers

## Abstract

The abilities of chemokines in orchestrating cellular migration are utilised by different (patho-)biological networks including malignancies. However, except for CXCR4/CXCL12, little is known about the relation between tumour-related chemokine expression and the development and progression of solid tumours like breast cancer. In this study, microarray analyses revealed the overexpression of chemokine CXCL13 in breast cancer specimens. This finding was confirmed by real-time polymerase chain reaction in a larger set of samples (*n*=34) and cell lines, and was validated on the protein level performing Western blot, ELISA, and immunohistochemistry. Levels of CXCR5, the receptor for CXCL13, were low in malignant and healthy breast tissues, and surface expression was not detected *in vitro*. However, we observed a strong (*P*=0.0004) correlation between the expressions of CXCL13 and CXCR5 in breast cancer tissues, indicating a biologically relevant role of CXCR5 *in vivo*. Finally, we detected significantly elevated serum concentrations of CXCL13 in patients with metastatic disease (*n*=54) as compared with controls (*n*=44) and disease-free patients (*n*=48). In conclusion, CXCL13 is overexpressed within breast cancer tissues, and increased serum levels of this cytokine can be found in breast cancer patients with metastatic disease pointing to a role of CXCL13 in the progression of breast cancer, suggesting that CXCL13 might serve as a useful therapeutic target and/or diagnostic marker in this malignancy.

Originally, chemokines (‘chemotactic cytokines’) and their receptors gained substantial scientific interest because of their central role in orchestrating immune responses by directing lymphocyte movement to the thymus, lymphoid tissues, and sites of inflammation (Luster, 1998). Recent evidence, however, has shown that the unique abilities of chemokines in regulating cellular migration are utilised by a much wider variety of (patho-)biological networks, including developing haematological malignancies and solid tumours (Balkwill, 2004).

The more than 40 human chemokines described are divided into four groups as follows (Bacon *et al*, 2002): the ‘CXC’ chemokines, defined by the presence of a single amino acid residue surrounded by two cysteines; the ‘CC’ chemokines, defined by two adjoined cysteine molecules; the ‘XC’ chemokines, defined by the loss of one cysteine residue; and the ‘C3XC’ chemokines (three interposed amino acids). Chemokines and their receptors are described by their family (e.g., CXC) combined with the letter R or L (‘receptor’ and ‘ligand’) and a member-specific number.

Although it was self-evident that proteins guiding lymphocyte migration and formation should be involved in the development of haematological malignancies – as has been demonstrated for T- and B-cell lymphomas (Fujii *et al*, 2002; Basso *et al*, 2004; Ek *et al*, 2006; Harasawa *et al*, 2006), multiple myeloma (Alsayed *et al*, 2007), and leukaemias (Voermans *et al*, 2002; Chunsong *et al*, 2006) – reports about the role of chemokines and their receptors in solid cancers have emerged only recently. For example, the expression of CXCR4 on melanoma cells was associated with increased rates of metastasis and patient mortality (Longo-Imedio *et al*, 2005), whereas another study suggests that interleukin-8 (IL-8) and its two chemokine receptors CXCR1 and CXCR2 might promote prostate cancer progression through autocrine signalling of prostate cancer cells (Murphy *et al*, 2005). Other authors found that CXCL12 might stimulate cell migration, cell growth, and invasion of ovarian cancer cells (Scotton *et al*, 2002), and that CXCR4 was found to be expressed on the majority of glioma cell lines studied and on patients' tissue samples. After adding the CXCR4 agonist CXCL12, glioma cell lines were prevented from apoptosis and showed increased chemotaxis (Zhou *et al*, 2002). A role of the chemokine/receptor pair CXCL12/CXCR4, normally controlling cell trafficking within the marrow (Nagasawa, 2000), in the development of solid tumour bone marrow metastases has been shown for small-cell lung cancer (Burger *et al*, 2003), breast cancer (Muller *et al*, 2001), and renal cell cancer (Zagzag *et al*, 2005). Accordingly, an entanglement of chemokine signalling pathways with tumour cell properties, such as proliferation, survival, adhesion, and chemotaxis, has been suggested (Laurence, 2006).

To gain insight into mechanisms by which chemokines might affect breast cancer development through local and microenviromental migration signalling, we analysed malignant and normal tissue samples from patients with breast cancer regarding their chemokine expression profiles.

CXCL13 is a chemokine ligand originally termed B-cell-attracting chemokine 1 (bca-1), which is known to be expressed by stromal cells within B-cell follicles in secondary lymphoid tissues (Gunn *et al*, 1998). It has a crucial function in germinal centre formation (Allen *et al*, 2004) through interaction with its receptor CXCR5 expressed on follicular B cells (Bowman *et al*, 2000). Under normal conditions, CXCL13 is furthermore expressed by follicular dendritic cells (Vissers *et al*, 2001), macrophages (Carlsen *et al*, 2004), and germinal centre T cells.

We found that CXCL13 is overexpressed within breast cancer tissues at the mRNA and protein levels and that increased serum levels of this cytokine can be found in breast cancer patients with metastatic disease pointing to a possible role of CXCL13 within the development and progression of breast cancer.

## Materials and Methods

### Patients, healthy blood donors, and breast cancer cell lines

A total number of 136 consecutive, consenting breast cancer patients were included in the study. Operable patients (*n*=34) were admitted to the Mammazentrum at the Jerusalem Hospital in Hamburg (Germany), and tumour samples as well as normal adjacent tissues were collected during surgery. Non-malignant tonsils were collected from consenting patients during routine tonsillectomy performed at the Department of Otorhinolaryngology at the University Medical Center Hamburg-Eppendorf (Germany). Tissues were stored in RNAlater (Ambion, Austin, TX, USA) at −80°C until further use. Serum samples were obtained from breast cancer patients receiving adjuvant treatment or chemotherapy for metastasised disease at the Department of Oncology/Hematology at the University Medical Center Hamburg-Eppendorf. Serum samples from 44 blood donors were obtained as controls. The study protocol had received approval by the local ethics committee.

### Breast cancer cell lines and short-term-activating culture

Human breast cancer cell lines, MDA-MB-231, MDA-MB-453, MDA-MB-468, MCF-7, ZR-75, BT-20, and CAMA-1, were kindly provided by the New York branch of the Ludwig Institute for Cancer Research. Cell lines were maintained in DMEM supplemented with 10% foetal bovine serum, 100 U ml^−1^ penicillin and streptomycin (Invitrogen, Carlsbad, CA, USA). For cytokine-induced activation, cell lines were cultured for 72 h in complete medium with or without activating cytokines TNF-*α* (25 ng ml^−1^; BD Bioscience, San Jose, CA, USA), INF-*γ* (100 U ml^−1^; R&D Systems, Minneapolis, MN), or both. Cells were harvested and were analysed for the expressions of CXCL13 and CXCR5 at baseline as well as after 24, 48, and 72 h.

### Reverse-transcription PCR

Total RNA was extracted from cell lines applying the RNeasy Mini Kit (Qiagen, Hilden, Germany) and from tissue samples using guanidium isothiocyanate for denaturation, followed by caesium chloride gradient ultracentrifugation overnight (36 000 r.p.m., 4°C). Reverse transcription was performed using AMV reverse transcriptase (Promega, Madison, WI, USA) and was run at 42°C for 45 min with heat inactivation of the enzyme at 95°C for 5 min. Polymerase chain reaction primers and conditions used for the analysis are as follows: CXCL13 forward primer (F): 5′-GAGGCAGATGGAACTTGAGC-3′; CXCL13 reverse primer (R): 5′-CTGGGGATCTTCGAATGCTA-3′; CXCR5-F: 5′-CTTCGCCAAAGTCAGCCAAG-3′; CXCR5-R: 5′-TGGTAGAGGAATCGGGAGGT-3′; GADPH-F: 5′-TGATGACATCAAGAAGGTGG-3′; and GADPH-R: 5′-TTTCTTACTCCTTGGAGGCC-3′. For PCR analysis of single genes, 4 *μ*l first-strand cDNA (equivalent to 0.1 *μ*g RNA) was amplified after the preparation of 25 *μ*l of PCR reaction mixtures containing transcript-specific oligonucleotides (10 pM), 2 U of AmpliTaq Gold (Perkin Elmer, Weiterstadt, Germany), 10 nM of each dNTP (dATP, dTTP, dCTP, and dGTP), and 1.67 mM MgCl_2_. After 35 PCR cycles, products were separated on 1.5% agarose gels, stained with ethidium bromide, visualised with ultraviolet light, recorded using a CCD camera, and assessed for expected size. Quality of cDNA was tested by reverse-transcription PCR (RT–PCR) using primers for housekeeping gene GAPDH. All RT–PCR experiments were performed at least twice. Negative controls without cDNA were integrated into all PCR reactions. To assess primer specificity, PCR products were analysed repeatedly by sequence analysis.

### Real-time PCR

Primer sequences for target genes used in real-time are the same as the ones used in conventional PCR. A master mix of the following components was prepared at the indicated final concentrations: 4.0 mM MgCl_2_, 400 nM forward and reverse primers, 200 nM dNTP (Invitrogen, Karlsruhe, Germany), 1% DMF, BSA at 250 *μ*g ml^−1^, SYBR Green I (Sigma, St Louis, MO, USA) diluted at 1 : 20 000, and 1 Unit FastStart Taq DNA Polymerase (Roche Diagnostics, Branchburg, NJ, USA) in a total volume of 20 *μ*l. Next, samples were analysed using a LightCycler (Roche Diagnostics). After an initial denaturation at 95°C for 10 min, PCR reactions were cycled 40 times as follows: 10 s at 95°C, 5 s at adequate annealing temperature (CXCL13 and CXCR5 at 61°C; GADPH at 61°C) and 15 s at 72°C (elongation). Fluorescence intensity was measured at the end of each elongation phase. A melting curve analysis was carried out immediately after amplification. A standard curve prepared of the PCR product cloned into a pcDNA2.1 vector using the TA cloning kit (Invitrogen, Karlsruhe, Germany) was prepared to determine the concentration of target transcripts in cDNA samples. Results are given as copy numbers of the target gene/1 × 10^6^ copies of housekeeping gene GAPDH.

### Real-time PCR array

A quantitative mRNA expression analysis of 84 chemokines/cytokines and their receptors was performed on 10 tumour tissue samples and 10 healthy breast tissue samples applying the chemokines/chemokine receptors RT2 profiler™ PCR Array (SuperArray, Frederick, MD). The following chemokine and related genes were analysed: chemokines: CCL1, CCL2, CCL3, CCL4, CCL5, CCL7, CCL8, CCL11, CCL13, CCL15, CCL16, CCL17, CCL18, CCL19, CXCL1, CXCL2, CXCL3, CXCL5, CXCL6, CXCL8, CXCL9, CXCL10, CXCL11, CXCL12, and CXCL13; chemokine receptors: CCR1, CCR2, CCR3, CCR4, CCR5, CCR6, CCR7, CCR8, CCR10, CCRL1, CCRL2, BLR1, CXCR3, CXCR4, CXCR6, and CYFIP2; other chemokines and related genes: AGTRL1, BDNF, C5, C5R1 (GPR77), CCBP2, CKLF, CKLFSF1, CKLFSF2, CKLFSF3, CKLFSF4, CMKLR1, CSF3, CX3CL1, CX3CR1, ECGF1, GDF5, GPR31, GPR77, GPR81, HIF1A, IL-13, IL-16, IL-18, IL-1A, IL-4, IL-8RA, LTB4R, MMP2, MMP7, MYD88, NFKB1, SCYE1, SDF2, SLIT2, TCP10, TLR2, TLR4, TNF, TNFRSF1A, TNFSF14, TREM1, VHL, XCL1, and XCR1. Extraction of RNA and reverse transcription were preformed as described above. PCR array analysis was performed according to the manufacturer's instructions using an iCycler System (Bio-Rad, Waltham, MA, USA). RNA expression of target genes was normalised for the expression of housekeeping gene 18S rRNA, and results were compared between malignant and normal samples according to the 2^−ΔΔ*C*t^ method. Results were considered significant if tumour-related relative mRNA expression was at least threefold higher or lower than that of autologous healthy tissue.

### Western blotting

Protein lysates were prepared from tumour samples, healthy breast tissues, cell lines, and non-malignant tonsils using standard lysis buffer containing a protease inhibitor cocktail (Sigma-Aldrich, St Louis, MO, USA) and were subsequently denaturated for 10 min at 70°C. Samples of lysates containing 30 *μ*g of total protein were resolved on 4–12% Bis-Tris SDS–PAGE gels (Invitrogen, Carlsbad, CA, USA) under reducing conditions. Proteins were blotted on Hybond-ECL nitrocellulose membranes (Amersham Biosciences, Piscataway, NJ, USA), blocked overnight at 4°C with Top-Block (Fluka, Buchs, Switzerland), and incubated with 1 *μ*g of primary antibody directed against CXCL13 (clone 53610; R&D Systems), CXCR5 (clone 51505; R&D Systems), and *β*-actin (clone SC-47778; Santa Cruz Biotechnology, Santa Cruz, CA, USA) for 4 h at room temperature. Next, secondary HRP-labeled anti-mouse monoclonal antibody (R&D Systems) was applied for 1 h at room temperature. Specific binding was visualised by chemiluminescence (ECL Western Blotting Analysis System, Amersham Biosciences).

### Flow cytometry

For the analysis of CXCR5 protein expression, cell lines were fixed using FACS Lysing Solution (BD Bioscience) and were permeabilised using Permeabilising Solution (BD Bioscience). For analysis of cell surface expression, cells were stained and then fixed; for cytoplasmatic staining, cells were fixed and permeabilised before staining with a PE-conjugated anti-CXCR5 antibody (Clone 51505, R&D Systems) or an appropriate isotype control. Samples were analysed using an FACSCalibur cytometer and CELLQuest software (BD Biosciences).

### Immunohistochemistry

Immunohistochemistry for CXCL13 and CXCR5 was performed on formalin-fixed, paraffin-embedded tissue sections, which had been obtained for routine diagnostics. Briefly, slides were deparaffinised and pretreated with 10 mmol l^−1^ citrate, pH 6.0 (Zymed, South San Francisco, CA, USA) in a steam pressure cooker (Decloaking Chamber; BioCare Medical, Walnut Creek, CA, USA) followed by washing in distiled water. All further steps were performed at room temperature in a hydrated chamber. The slides were pretreated with peroxidase block (Dako, Glostrup, Denmark), followed by blocking with goat serum diluted at 1 : 5 in 50 mmol l^−1^ Tris-HCl, pH 7.4 for 20 min. Primary murine anti-human CXCL13 (clone 53610) and CXCR5 (clone 51505; both R&D Systems) antibodies were applied at a 1 : 10 dilution in 50 mmol l^−1^ Tris-HCl, pH 7.4 with 3% goat serum for 1 h. The slides were washed in 50 mmol l^−1^ Tris-HCl, and goat anti-mouse horseradish peroxidase-conjugated antibody (Envision detection kit; Dako) was applied for 30 min. After further washing, immunoperoxidase staining was performed using a Diaminobenzidine Chromogen Kit (Dako), as per the manufacturer's instructions, and the slides were counterstained with haematoxylin.

### Enzyme-linked immunosorbent assay

Serum concentrations of soluble CXCL13 protein were determined using a commercially available Quantikine kit (R&D Systems) according to the manufacturer's instructions. After development of the enzyme-linked immunosorbent assay (ELISA) plates, absorbance was read at 450 nm using a spectrophotometer (SLT Labinstruments, Salzburg, Austria). The concentration of CXCL13 in the sera was interpolated from a standard curve, which was generated using the respective recombinant protein.

### Statistical analysis

The Wilcoxon test was applied to results from tumour samples *vs* autologous healthy tissues, whereas the Mann–Whitney *U*-test was used to examine differences between samples of tumour patients and controls. Spearson's rank test was used to analyse correlations between gene expression and clinicopathological characteristics of the patients. Statistical analysis was performed using SPSS software (SPSS Inc., Chicago, IL, USA). Results were considered significant with *P*<0.05.

## Results

### Microarray analysis demonstrates strong CXCL13 overexpression in breast cancer tissue as compared with normal breast tissue

**Figure 1:**
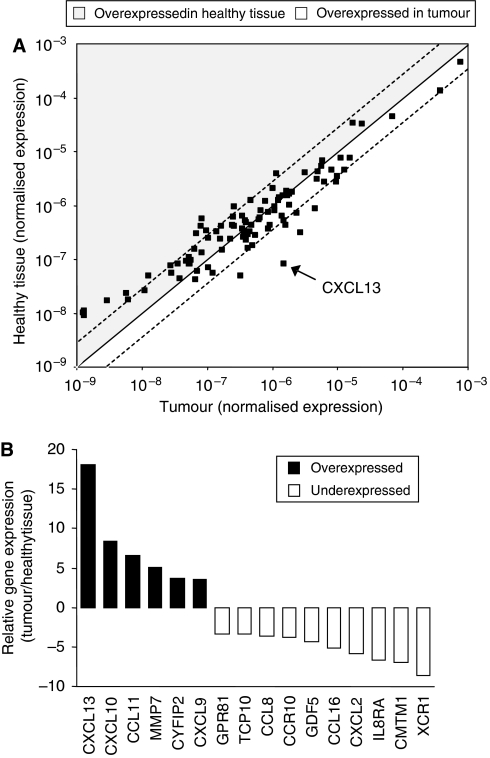
CXCL13 is the most strongly overexpressed chemokine in breast cancer tissue as compared with normal breast tissue. Using a real-time PCR-based and pathway-focused microarray analysis on tissue samples from 10 patients whose tumours had been surgically removed, we simultaneously analysed mRNA expression levels of 84 chemokines/cytokines and their receptors in malignant and autologous healthy breast tissue. All patients included were stratified according to their status of disease (T_2_N_0_M_0_, stage II). (**A**) Dots represent mean expression of single genes in malignant and healthy tissues after normalisation for housekeeping gene 18S rRNA. Dotted lines indicate the arbitrary cutoff value of threefold over- or underexpression in malignant *vs* normal tissues. (**B**) The microarray analysis revealed 16 target genes that showed at least three times higher or lower mRNA expression levels in malignant than in autologous healthy breast tissue. Black columns represent genes upregulated in tumours and white columns indicate genes that were downregulated as compared with normal breast tissue.

Given the increasingly important role of chemokine/chemokine receptor interactions in the development and spreading of various malignancies, we aimed at applying a screening approach to look for chemokines that might have significant functions in the biology, and possibly in the pathophysiology, of breast cancer. We therefore performed a real-time PCR-based and pathway-focused microarray analysis on malignant and autologous healthy breast tissue samples from 10 patients whose tumours had been surgically removed. All patients included showed the same status of disease (T_2_N_0_M_0_; stage II). Simultaneously examining mRNA expression levels of 84 chemokines/cytokines and their receptors in malignant and non-malignant tissue, we found 16 target genes that were differently expressed in breast cancer tissues as compared with their normal counterparts (Figure 1A and B). Six genes were significantly higher expressed in tumour tissue than in healthy tissue. Of these, chemokine CXCL13 was by far the most strongly and consistently overexpressed gene with a mean expression level in tumour samples that was 18 times higher than the one observed in autologous benign breast samples (Figure 1B).

### CXCL13 is consistently overexpressed at the RNA and protein levels in tumour samples of breast cancer patients

**Figure 2:**
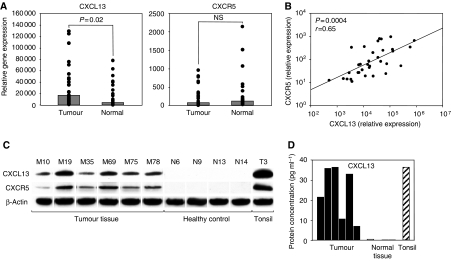
CXCL13 is consistently overexpressed in tumours of breast cancer patients. (**A**) Tumours and non-malignant breast tissues of 34 patients with breast cancer were analysed regarding the expression levels of CXCL13 and its receptor CXCR5 applying real-time PCR and results were normalised to the expression levels of housekeeping gene GAPDH. Black dots represent copy numbers of the target gene for each benign or malignant sample, respectively, and bars represent medians calculated. Benign and malignant samples were compared using Wilcoxon's test. (**B**) Performing a correlative analysis of CXCL13 and CXCR5 RNA expressions in breast cancer samples, a significant association between expression levels of both genes was observed. (**C**) Lysates of six tumour samples, four healthy breast tissues, and one non-malignant tonsil were analysed by Western blot for the protein expressions of CXCL13 and CXCR5. (**D**) To confirm findings obtained in the Western blot analysis, an ELISA was performed quantifying the absolute concentration of CXCL13 protein in lysates consisting of whole breast cancer protein.

To confirm our findings obtained by the RT–PCR array, we applied a CXCL13-specific real-time PCR to a larger set of samples from 34 breast cancer patients comprising all stages of disease (Table 1). Performing a comparative analysis of malignant and autologous healthy breast tissues, we observed readily detectable levels of CXCL13 in malignant as well as in non-malignant samples (Figure 2A). Comparing CXCL13 mRNA expression levels of malignant and normal breast tissue samples, however, we found that CXCL13 mRNA levels were significantly elevated in the malignant samples, confirming that increased expression of this chemokine is indeed a feature distinguishing tumour-infiltrated breast tissues from their healthy counterparts. Examining the presence of CXCR5, which is the only known receptor for chemokine CXCL13, within breast cancer tissues, we detected only a comparably weak expression (Figure 2A). We also did not observe any significant correlations between tumour RNA expression of CXCL13 or CXCR5 and clinical characteristics of individual patients (data not shown). However, we detected a strong and significant (*P*=0.0004) correlation between copy numbers of both genes analysed within breast cancer tissue (Figure 2B), indicating a biologically relevant role of CXCR5 as a receptor for CXCL13 in this solid tumour.

Performing Western blot analyses of CXCL13 and CXCR5 expressions on lysates of six randomly selected breast cancer samples and four healthy breast tissue samples, we observed a weak but constantly detectable expression of the chemokine receptor CXCR5 in tumour samples, whereas protein expression of this chemokine receptor was not detectable in the healthy samples analysed (Figure 2C). In accordance with RNA expression levels, CXCL13 protein was commonly found in tumour samples, whereas it was absent from all healthy breast tissues analysed (Figure 2C). Marked overexpression of CXCL13 protein in breast cancer tissue was further confirmed performing a quantitative ELISA analysis with the same lysates of tumours and healthy controls. Remarkably, in this quantitative assay, protein concentrations of CXCL13 in breast cancer samples almost approached the levels found in non-malignant inflamed tonsils, which served as a positive control (Figure 2D).

### CXCL13 and CXCR5 are expressed intracellularly in breast cancer cell lines but are not detectable on the cell surface

**Figure 3:**
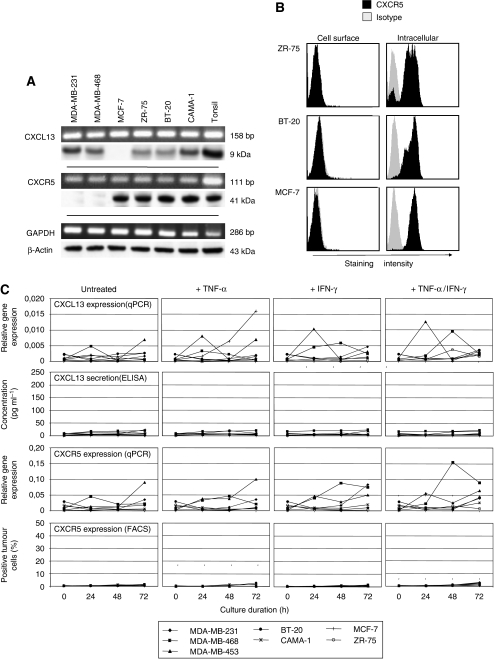
CXCL13 and its receptor CXCR5 are expressed intracellularly in breast cancer cell lines. (**A**) Expressions of chemokine CXCL13 and its receptor CXCR5 were examined in six breast cancer cell lines applying conventional RT–PCR (upper rows) and Western blot (lower rows). Housekeeping genes GAPDH and *β*-actin served as internal controls. (**B**) Breast cancer cell lines ZR-75, BT-10, and MCF-7 were examined regarding the protein expression of CXCR5 using flow cytometry. Histograms indicate staining intensity applying anti-CXCR5 antibody (black) or an appropriate isotype control (grey). (**C**) Seven breast cancer cell lines were cultured for 72 h in complete medium with or without activating cytokine TNF-*α* or INF-*γ*. mRNA levels of CXCL13 and CXCR5 were evaluated at baseline as well as after 24, 48, and 72 h applying real-time PCR. At the same time points, the concentration of CXCL13 protein in the culture supernatant was quantified using an ELISA. Cell surface expression of CXCR5 protein was evaluated by flow cytometry.

Given the significant overexpression of CXCL13 at the mRNA and protein levels in breast cancer tumour samples, we sought to determine the expression patterns of the chemokine ligand and its receptor in human breast cancer cell lines. We analysed six different breast cancer cell lines and found RNA and protein expressions of CXCL13 in all but one and CXCR5 expression in four of six cell lines (Figure 3A). Analysing the breast cancer lines by flow cytometry, we failed to detect a significant surface expression of the chemokine receptor, however, by performing intracellular staining, we observed strong expression of CXCR5 in the cytoplasma of cell lines ZR-75, BT-20, and MCF-7 (Figure 3B).

Technical difficulties, mainly due to unspecific intracellular staining, did not allow for accurate analysis of CXCL13 expression by flow cytometry (data not shown). Therefore, we performed extensive analyses of CXCL13 protein concentrations in the supernatant of breast cancer cell lines that had been cultured for 72 h under different conditions to find shed CXCL13. However, culturing of cell lines with or without the addition of activating cytokine TNF-*α* or INF-*γ* did not result in the detection of CXCL13 in the supernatant (Figure 3C), whereas intracellular CXCL13 mRNA was consistently detectable using real-time PCR, independently from the addition of cytokines. Accordingly, CXCR5 mRNA was consistently detectable using real-time PCR, but the tumour cells remained negative for surface expression of the protein even after the addition of activating cytokines (Figure 3C).

### Epithelial tumour cells of patients with breast cancer show immunohistochemically detectable overexpression of CXCL13

**Figure 4:**
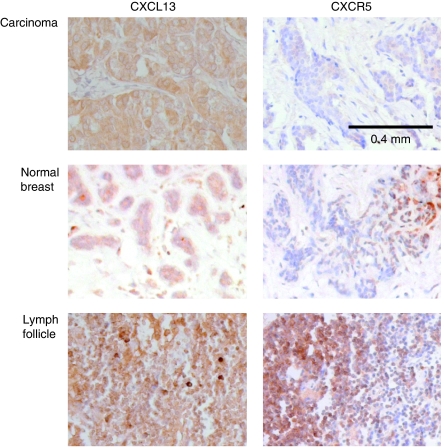
Immunohistochemistry localises the overexpression of CXCL13 to epithelial tumour cells in tissue samples of breast cancer patients. Immunohistochemical staining using appropriate anti-CXCL13 and anti-CXCR5 antibodies was performed on 10 paraffin-embedded malignant or non-malignant tissue samples, respectively. Non-malignant lymph follicles removed from breast tissue served as a positive control for CXCL13 and CXCR5 stainings and showed homogenous staining throughout the node.

To further validate our findings of CXCL13 protein overexpression in human breast cancer and to localise the expression within a given tissue sample, we performed immunohistochemistry on breast cancer samples and autologous breast tissue samples of the same patients using lymph follicles as positive controls. Importantly, we did not observe significant numbers of tumour-infiltrating leukocytes expressing CXCL13 or CXCR5. However, we found a strong cytoplasmatic expression of CXCL13 in epithelial tumour cells, which was comparable with levels found in lymph follicles of draining lymph nodes from the same patients determined to be free of tumour (Figure 4). Benign epithelial cells also expressed CXCL13, however, to a much lesser extent. CXCR5 staining of the same specimens revealed a weak cytoplasmatic staining of epithelial cells with comparable levels between malignant and benign samples.

### Levels of CXCL13 protein are increased in the serum of breast cancer patients

**Figure 5:**
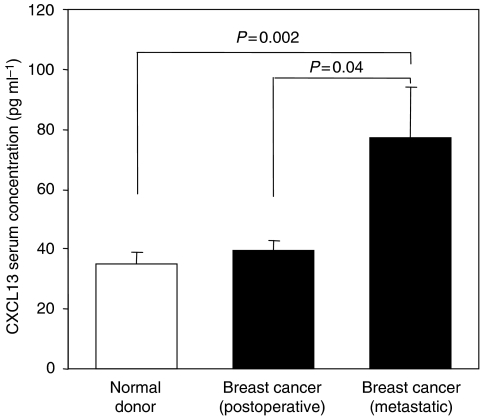
Serum concentrations of CXCL13 are elevated in breast cancer patients with metastatic disease. Serum concentrations of CXCL13 were analysed by ELISA in 44 healthy blood donors (white bar), 48 breast cancer patients without evidence of disease after surgical resection of their tumour and 54 patients with metastatic breast cancer (black bars). Bars show mean concentrations of CXCL13 plus standard deviations. Results were compared between groups applying the Mann–Whitney *U*-test.

Breast cancer cell lines might not reflect the biological behaviour of original tumour tells and certain features, such as chemokines secretion or release might diminish or wear off during cell culture and repeated passages. Therefore, we next asked the question whether soluble CXCL13 protein could be detected in the *in vivo* setting, given the high levels of protein expression within breast cancer tissue samples as shown above. To this end, we analysed serum concentrations of CXCL13 protein in 44 healthy blood donors, 48 breast cancer patients without evidence of disease after surgical resection of their tumour, and 54 patients with metastatic breast cancer. Although there was no difference in CXCL13 serum concentrations between the healthy control group and patients without evidence of disease (Figure 5), we found significantly elevated serum concentrations of CXCL13 in patients with metastatic disease as compared with normal controls and with breast cancer patients without evidence of disease after surgical removal of their original tumour.

## Discussion

Besides our current report, there are very limited data on CXCL13 or CXCR5 expression in non-migratory cells, let alone in solid tumours. In one report, immunohistochemistry revealed CXCR5 expression in a significant proportion of human colon carcinoma specimens (Gunther *et al*, 2005), whereas another group demonstrated cytoplasmatic expression in pancreatic cancer cells and cell lines (Meijer *et al*, 2006). We show here for the first time that the ligand for CXCR5, CXCL13, is the most significantly overexpressed chemokine in breast cancer, supporting the idea of a role of CXCL13/CXCR5 interactions in promoting initiation and/or progression of this tumour type and, possibly, other human cancers. Furthermore, given the elevated CXCL13 expression in the serum of patients with metastatic disease as compared with patients without evidence of tumour burden, this chemokine may also serve as a tumour marker.

In our analysis of breast cancer cell lines and tumour samples, we found the expression of chemokine receptor CXCR5 to be restricted to the cytoplasma, a finding that is in line with observations of Muller *et al* (2006) who detected CXCR5 intracellulary but did not detect surface expression of CXCR5 in cell lines and tumour samples from patients with metastatic head and neck cancer. Although these findings, at first glance, do not support the concept of CXCR5 as the main target for CXCL13 overexpression in breast cancer, we believe that they do not reflect the potential role this chemokine receptor might play *in vivo*. It may very well be that tumour cells express surface CXCR5 only during certain stages of tumour organisation and internalise the receptor after arrival within an area of maximum concentration of ligand CXCL13. This idea is supported by the findings of others who observed that, in the case of chronic lymphatic leukaemia, CXCR5 and its ligand were overexpressed in malignant B cells but that CXCR5 was downregulated after stimulation with soluble CXCL13 (Burkle *et al*, 2007). Importantly, a concentration gradient- and time-dependent downregulation would also explain our observation of a strong correlation between CXCL13 expression and the presence of its receptor within breast cancer tissues despite the lack of CXCR5 overexpression in the same tissues.

In addition, the hypothesis that an externalisation of receptor CXCR5 is restricted to certain phases of cancer development *in vivo* is also supported by the findings of Meijer *et al* (2006) who did not detect CXCR5 expression on a variety of tumour cell lines cultured *in vitro* but who found this chemokine receptor to be expressed on the surface of the same cells several days after injection into mice.

What might the biological role of CXCR5/CXCL13 interactions be in the case of human cancers? Our finding of increased levels of soluble CXCL13 protein in the peripheral blood of breast cancer patients with advanced disease clearly suggests that this chemokine might be involved in the process of metastasisation. One might imagine at least three possible ways for CXCL13 to mediate a promoting effect on tumour development, the first two of them involving cellular immunity. First, it could be that breast cancer cells produce and release chemokines to allure cells, such as lymphocytes, monocytes, or dendritic cells, that are capable of secretion of cytokines directly promoting tumour cell growth and survival. Such an effect has been demonstrated for Hodgkin's lymphoma (Teruya-Feldstein *et al*, 1999; Buri *et al*, 2001). Another possibility might be that CXCL13 mediates some form of protection against immune-mediated anti-tumour immunity. Such a phenomenon has been reported for leukaemias, where CXCL13-expressing malignant B cells showed an increased resistance against TNF-*α*-mediated apoptosis (Qiuping *et al*, 2005; Chunsong *et al*, 2006).

Our observation of a very low frequency of CXCL13-expressing leukocytes present within breast cancer tissue, however, would argue against the latter two hypotheses. Therefore, we favour the third possible explanation, suggesting that breast cancers cells might supply themselves with a growth advantage by the expression of CXCR5 and the release of CXCL13 *in vivo*. This view is also supported by a recent study demonstrating a growth advantage of CXCR5-positive cancer cells within the liver of mice presumably through CXCL13 produced by liver cells in their microenvironment (Meijer *et al*, 2006).

In our view, the most perspicuous explanation for the increased presence of CXCL13 in breast cancer is that CXCR5/CXCL13 interactions might function in a similar way as they do in lymphatic tissue. Breast cancer cells might interact through CXCR5/CXCL13 interactions to organise cellular cluster formation and compartmentalisation as has been shown for B cells in renal allografts (Steinmetz *et al*, 2005) and in salivary glands of patients with Sjogren's syndrome (Barone *et al*, 2005). This way, CXCL13 and its ligand might contribute significantly to tumour formation, and therapeutic interventions aiming at interrupting CXCL13/CXCR5 interactions might be of benefit for the clinical course of patients with breast cancer.

It has recently been shown (Burkle *et al*, 2007) that the stimulation of leukaemic B cells with CXCL13 results in a prolonged activation of p44/42 mitogen-activated protein kinases. Interestingly, p44/42 mitogen-activated protein kinase activation has also been shown to be involved in oncogenic transformation of epithelial breast cancer cells and their protection from oxidative stress (Zhu *et al*, 2005; Mohankumar *et al*, 2007). Therefore, we suggest that this kinase pathway represents one possible route through which CXCL13/CXCR5 interactions might exert their biological function on a molecular level. Unfortunately, the lack of CXCR5 surface expression on breast cancer cell lines did not allow for consequent functional analyses of the role of CXCL13 in breast cancer *in vitro*. Further studies, however, are underway in our laboratory examining the exact consequences of CXCL13/CXCR5 interactions for the development and/or progression of breast cancer *in vivo*.

## Figures and Tables

**Table 1:** Clinicopathological characteristics of breast cancer patients analysed for CXCL13/CXCR5 expression by real-time PCR

**Characteristics**	**Number of patients per group**
Total	34
Age (median and range) years	63 (43–85)
	
*pT status*	
pT_1_	13
pT_2_	19
pT_3_	0
pT_4_	1
	
*pN status*	
pN_0_	20
pN_1_	9
pN_2_	3
pN_3_	2
	
*M status*	
M_0_	30
M_1_	2
	
*Stage*	
I	10
II	15
III	4
IV	2
	
*Grading*	
Well differentiated	4
Moderately differentiated	21
Poorly differentiated	8

A total of 34 patients with breast cancer were classified according to clinical features and pathological characteristics of their tumour samples. Assessment of T status and grading was only available for 33 patients, M status and classification into stages of the disease for 32 and 31 patients, respectively.
